# New Insights to Design Electrospun Fibers with Tunable Electrical Conductive–Semiconductive Properties

**DOI:** 10.3390/s23031606

**Published:** 2023-02-01

**Authors:** William Serrano-Garcia, Irene Bonadies, Sylvia W. Thomas, Vincenzo Guarino

**Affiliations:** 1 Advanced Materials Bio & Integration Research (AMBIR) Laboratory, Department of Electrical Engineering, University of South Florida, Tampa, FL 33620, USA; 2 Institute of Polymers, Composites and Biomaterials, National Research Council of Italy, Via Campi Flegrei 34, 80078 Pozzuoli, Italy; 3 Institute of Polymers, Composites and Biomaterials, National Research Council of Italy, Mostra d’Oltremare, Pad.20, 80125 Naples, Italy

**Keywords:** fiber electronics, conductive fiber-based structures, electrospinning, biointegration, semiconducting polymers, conducting polymers

## Abstract

Fiber electronics, such as those produced by the electrospinning technique, have an extensive range of applications including electrode surfaces for batteries and sensors, energy storage, electromagnetic interference shielding, antistatic coatings, catalysts, drug delivery, tissue engineering, and smart textiles. New composite materials and blends from conductive–semiconductive polymers (C-SPs) offer high surface area-to-volume ratios with electrical tunability, making them suitable for use in fields including electronics, biofiltration, tissue engineering, biosensors, and “green polymers”. These materials and structures show great potential for embedded-electronics tissue engineering, active drug delivery, and smart biosensing due to their electronic transport behavior and mechanical flexibility with effective biocompatibility. Doping, processing methods, and morphologies can significantly impact the properties and performance of C-SPs and their composites. This review provides an overview of the current literature on the processing of C-SPs as nanomaterials and nanofibrous structures, mainly emphasizing the electroactive properties that make these structures suitable for various applications.

## 1. Introduction

**Figure 1:**
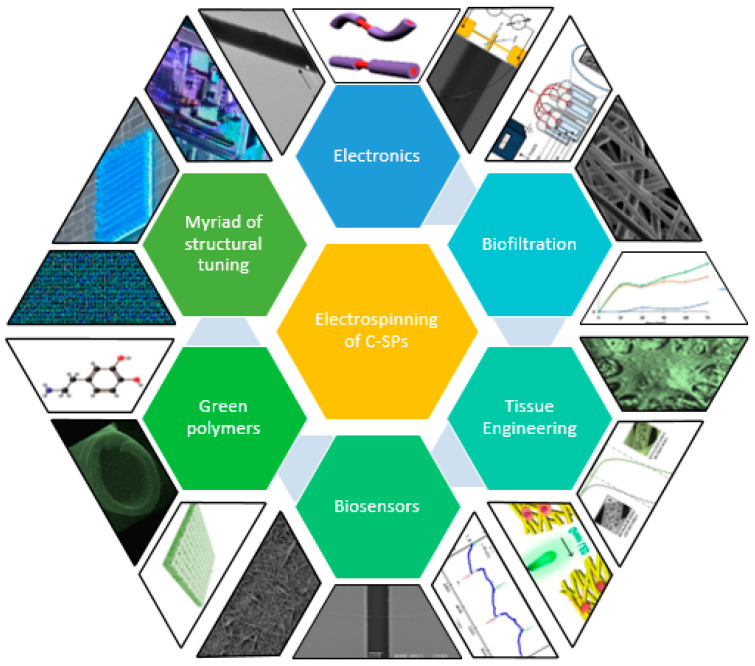
Electrospun nanofibers containing C-SPs: main applications for electronic devices and biological purposes.

Conductive nanofiber structures, such as those formed by the electrospinning technique, have been widely used in the fabrication of electrodes and electronic devices, receiving increased attention in recent years [1,2,3,4]. These structures have a wide range of potential applications including supercapacitors [5], sensors [6,7], energy storage [8], electromagnetic interference (EMI) shielding [9,10,11,12], environmental monitoring [13], biomedical diagnostics [14], catalysts for chemical reactions [15], and smart textiles for wearable electronics [16], health trackers [17] and smart clothing [18,19]. New composites and blends are being explored for the manufacture of such devices that are directly applied in the biomedical field as filters, active cell grown media, and biosensing. These nanostructures could achieve complex shapes, with a predefined spatial arrangement, large surface area-to-volume ratio, and high density [20,21,22]. Furthermore, electroactive polymers could be classified as conducting or semiconducting polymers due to its electronic behavior under various arrangements and doping [23]. These nanostructures made of conductive–semiconductive polymers (C-SPs) and other composites enable the creation of smart nanopatterns for the production of nanodevices in energy, electronics, the environment, and healthcare, which takes advantage of the structurally high surface area-to-volume ratios which enhance the tunability of the device and their application (Figure 1) [24].

C-SPs nanostructures have become prominent as transformative platforms for controlled drug delivery, bio and chemical sensing, separation and filtration membranes, wound healing, electrodes, p-n junctions/diodes, and FETs (field effect transistors), owing primarily to properties of an electrical, plasmonic, structural, optical, and mechanical nature. The versatility of these C-SPs materials to be casted into foams, thin films, gels, and extruded as fibers and yarns has increased the interest of the scientific and technical community. As these materials are processed, nanopatterned surfaces are developed to provide numerous advantages in the fabrication and functionality of nanostructures for nanodevices. These materials and subsequent engineered structures (i.e., polymer blends, post-processing) exhibit great potential in already known areas such as neural probes due the ability to modify the C-SPs behavior in order to match specific functions that require compatibility with organic tissue, achieving applications such as localized active drug delivery, among others. Pristine and composite C-SPs have distinct properties such as mechanical flexibility, electronic transport behavior, biocompatibility, chemical orthogonality, and optical interactions, making them promising candidates for use in the healthcare industry, energy sector, electronic platforms, and environmental field. Adaptable processing systems, doping tunability, versatile morphologies, and nano-inspired surfaces of C-SPs and their composites have been shown in studies to have a significant impact on properties and performance [25,26]. Furthermore, recent investigations have shown the ability of applying these polymers in key areas such as organic electronics, molecular filtration, and neuroscience.

As innovative and transformative devices and structures are being created using specified types of conducting polymers, such as poly(3-hexylthiophene-2,5-diyl) (P3HT), polyacetylene (PA), polypyrrole (PPy), polyaniline (PANI), poly(3,4-ethylenedioxythiophene) (PEDOT), polythiophene (PT), poly(phenylene) (PP) oxide and vinylene, poly(benzimidazobenzophenanthroline) (BBL), in their pure form and as composites, this paper provides a brief review of the literature on the processing of C-SPs and material properties that make these structures advantageous for current and future nanodevice applications. Furthermore, some of the most recent research advances in sensing and biomedical applications are discussed as well as an outlook.

## 2. The Electrospinning Technique

**Figure 2:**
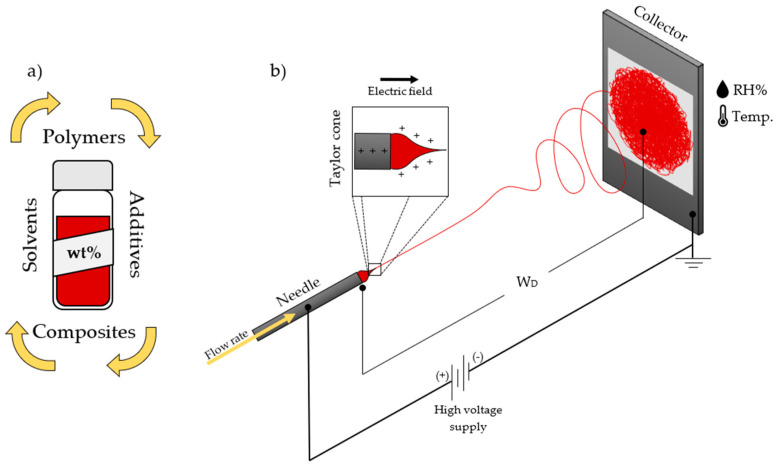
Schematic of fundamental steps that characterize the preparation of electrospun fibers via electrospinning technique. Solution preparation (**a**) and spinning essential variables (**b**).

The electrospinning technique is the most effective process to ensure the fabrication of engineered nanofibers with many papers going into the details of their formation [27,28,29]. Currently, there are commercially available modules and industrial systems that perform the electrospinning process with great precision, accuracy, and reliable results; however, the technique is simple and straightforward enough to essentially require three main components: a high-voltage power supply, a solution container (syringe for needle-based or an open surface container for needle-less techniques), and a collector where the extruded material will be deposited (Figure 2). Generally, this will offer the user a ready-to-spin setup. To be more precise, the high-voltage power supply has a range of 1 to 30 kV (while usually the 5 to 20 kV range works for most approaches), while a syringe pump for solution flow rate control and a conductive plate or a rotative drum of aluminum or stainless steel are used. Commonly, the positive charge is applied to the needle and the negative charge is applied to the collector. When a high voltage (~1.0 ± 0.5 kV/cm) is applied between the needle and the collector, at a desired working distance (W_D_), a strong electric field is formed. The solution is inserted into the electric field by means of a needle, promoting the droplet to positively charge and deform with further increasing the voltage. This increase in voltage promotes the formation of the Taylor cone (at the tip of the needle) and the formation of a charged jet at a critical voltage where the surface tension of the droplet is decreased enough to stretch the solution toward the collector. During the solution extrusion, it goes from a linear flow to a turbulent stretch. In this step, the solution jet enhances the solvent evaporation while decreasing its diameter and therefore solidifying, concluding the formation of the nanofibers. A straightforward summary of the electrospinning process involves the following steps.

- ***Preparation of the polymer solution and composites***: The first step in electrospinning is to prepare a polymer solution that is suitable for spinning into fibers. This involves dissolving the polymer in a suitable solvent or mix of solvents. The concentration of the polymer in the solution, as well as the choice of solvent, can affect the properties of the resulting fibers [30,31,32]. Parameters such as the viscosity [33], molecular weight [34], permittivity [35], and surface tension [36] will define the physical and chemical properties of the nanofibers.
- ***Environment conditioning***: At this step, the environmental conditions need to be controlled and stabilized for a reliable formation of nanofibers. Changes in temperature and humidity will affect the morphology and properties of the nanofibers [37,38].
- ***Electric field application***: The next step is to apply an electric field to the polymer solution. This is typically completed by using a high-voltage power supply and two metal electrodes, one of which is placed near the nozzle or spinneret and the other is placed at the W_D_. The electric field causes the polymer solution to be attracted to the electrode near the nozzle, which results in a thin jet of polymer being formed [39].
- ***Spinning of the fibers***: As the jet of polymer is attracted to the electrode near the nozzle, it is also subjected to the force of the electric field, which causes it to stretch and thin out. The jet is spun around a central axis as it is drawn toward the electrode, resulting in the formation of fibers. The diameter of the fibers is determined by the strength of the electric field, the viscosity of the polymer solution, the distance between the electrodes, and the solution flow rate [40].
- ***Collection of the fibers***: The fibers produced by electrospinning are typically collected on a substrate or frame placed near the electrode opposite the nozzle. The fibers can be collected as a nonwoven mat or as a continuous yarn, depending on the desired application [40].

The formation and characteristics of the nanofibers can be skillfully controlled by adjusting the solution concentration, the solvents (i.e., solubility, boiling point), the polymer properties (molecular weight, electrical properties), the parameters in the process (needle or needle-less, working distance, voltage, flow rate, collector type) as well in the surrounding environment (temperature *T*, humidity *RH%*, air flow).

There are several advantages to using electrospinning to produce fibers. First, it allows producing fibers with diameters in the nanometer to micrometer range, which is difficult to achieve using other techniques [41]. Second, it allows producing fibers with a high surface area to volume ratio, which can be useful for applications such as drug delivery and tissue engineering. Third, it is a relatively simple and inexpensive process, making it suitable for large-scale production. However, there are also some general limitations to the use of electrospinning. One constraint is that it can be difficult to control the properties of the fibers produced, such as their diameter, strength, and uniformity when the initial variables are not carefully controlled or totally ignored. Another limitation is that it can be difficult to spin certain polymers, such as those that are highly viscous or have a low melting point. Additionally, the fibers produced by electrospinning from polymers with low molecular weight are often fragile and prone to breakage, which can be a challenge for certain applications.

Despite these limitations, electrospinning has emerged as a versatile and widely used technique for the production of ultrafine fibers and nanofibers. It has the potential to revolutionize a wide range of industries and has already been used to develop a variety of innovative products, including filters, wound dressings, drug delivery systems, and energy storage devices.

## 3. Conductive and Semi-Conductive Polymers (C-SPs)

Even nowadays, most of the public sees polymers as non-conductive materials that are found in applications where electrical insulators are required. However, as recent as the beginning of the new millennium, the 2000 Nobel prize was awarded to the discovery and characterizations of conducting polymers, or in other words, “synthetic metals” or “electrically conductive plastics” [42]. The conducting polymer was polyacetylene, which was highly doped with bromine, increasing the conductivity of the material. While in metals, the valence electrons of a half-filled band (“sea of electrons”) are available to conduct electricity, in conducting polymers, such as polyacetylene, the electrons of conjugated double bonds act as the available charge to facilitate the electrical conductivity. Furthermore, the alternating single and double bonds between the molecules promote mechanical stability while the delocalization of π bonds acts as the charge carrier. This delocalization paves the way to the generation of charge carriers, which are responsible for increasing the conductivity of the material from an insulating to a conducting one [43].

Conductivity is a measure of a material’s ability to conduct electricity. It is typically expressed as a value of electrical conductivity (S/cm) or electrical resistivity (Ω cm). The lower the value, the more conductive the material is. Furthermore, the electrical conductivity of a polymer is dependent upon several factors, including its chemical structure, the degree of conjugation, the degree of crystallinity, and the presence of dopants or other additives. In general, conductive polymers have higher electrical conductivities than semiconductive polymers, which have electrical conductivities that are intermediate between those of metals and insulators. The electrical conductivity of a particular polymer can range from less than 1 S/cm for some undoped polymers to several thousands of S/cm for highly conductive polymers. For comparison, the electrical conductivity of aluminum, gold, copper, and silver is around 3.8 × 10^7^, 4.1 × 10^7^, 5.8 × 10^7^, and 6.3 × 10^7^ S/cm, respectively, while C-SPs range around 10^−12^–10^6^ S/cm [44].

It is worth noting that the electrical conductivity of a polymer can vary significantly depending on the type of polymer and the conditions under which it is measured. For example, the electrical conductivity of polyaniline can range from around 10^−2^ to 10^3^ S/cm [45,46], depending on the synthesis method, type of dopant, and the degree of doping [47]. Similarly, the electrical conductivity of polypyrrole can range from around 10^−9^ to 10^2^ S/cm [44,45,48]. Poly(3-hexylthiophene), or P3HT, is typically in the range of 10^−3^ to 10^2^ S/cm, while BBL is a highly conductive polymer, with electrical conductivities that are typically in the range of 10^−7^ to 10^1^ S/cm [49]. This is significantly higher than the electrical conductivity of most other polymers, which are typically in the range of 10^−3^ to 10^2^ S/cm. Polythiophene and polyphenylene oxide have a conductivity that ranges ~10^4^–10^6^ S/cm.

From an electrical perspective, there are three conventional groups of polymers utilized in the production of nanofibers: insulators, semiconductors, and conductive polymers.

Insulators are frequently used to make filters, biodegradable and biocompatible membranes for bone scaffolds and wound healing, and applications involving drug delivery [50]. Insulating polymers had been also used to ensure and support the formation of nanofibers by adding mechanical stability to materials such as oxide clusters, particle composites, metals and other polymers that are unable to form fibers on their own generally due to their low molecular weight. Indeed, these materials can be agglomerated into nanoparticles, nanodots, clusters, or crystals due to the low covalent structural interactions, forming composite nanofibers. Among insulators, the most used polymers used to ensure the formation of nanofibers includes the thermoplastics such as polystyrene (PS), polycaprolactone (PCL), polylactic acid (PLA), polyethylene oxide (PEO), polymethyl methacrylate (PMMA), and polyvinylidene difluoride (PVDF).

Semiconducting polymers are widely used and necessary for applications such as diodes, LED sensors, transistors, and solar cell configurations. Polymers with semiconductive properties have a lower conductivity than conductive polymers but are still able to conduct electricity to some extent. They have an electrical resistance that is higher than conductive polymers but lower than insulating polymers. Due to their ability to be blended with polymers that are biocompatible and encourage cell proliferation, some of these materials have also been studied for active drug delivery [51] and monitored cell cultures [52]. However, because of their typical low molecular weight, these polymers tend to form weak fibers during the formation of the charged jet during the electrospinning. Therefore, to provide the essential mechanical stability for fiber formation, homogeneous blends with high molecular weight insulating polymers are frequently used. Simultaneously, the insulating polymer might be incapable of forming nanometric fibers; therefore, the addition of a small quantity of a semiconducting or conducting polymer increases the solution’s charge and transforms the insulating polymer structure into an electroactive composite encouraging the formation of nanofibers at lower weight percentages with smaller fiber diameters. Indeed, the semiconducting polymer increases the solution’s conductivity, resulting in nanofibers that are thinner than those made with the nonconductive polymer alone. Examples of this type of polymer includes the classic sulfur-based p-type polymer, poly(3-hexylthiophene-2,5-diyl) (P3HT), and the nitrogen-based n-type ladder polymer, poly(benzimidazobenzophenanthroline) (BBL).

Conductive or conjugated polymers require higher doping doses to make them conduct electricity with high efficiency. They have a low electrical resistance and can carry electrical current just as effectively as some metals [53,54]. Examples of conductive polymers include the salt-based semi-flexible rod polymer polyaniline (PANI), doped polyacetylene (PA), branched poly (3,4-ethylenedioxythiophene) polystyrene sulfonate (PEDOT:PSS), polypyrrole (PPy), and the rigid-rod poly(p-phenylene vinylene) (PPV) (Table 1). Considering the conductivity range of C-SPs, these polymers can be included into the electroactive polymer’s regime. However, in this instance, an electroactive system is a fiber or composite fiber with the ability to conduct electricity through the incorporation of a semiconductive or conducting polymer.

## 4. Processing C-SPs via Electrospinning

To process C-SPs via electrospinning, it is possible to start from intrinsically conductive solutions, which includes conducting and semiconducting polymers, and nonconductive solutions that serve to realize the support for the conductive material which makes the system an electroactive composite. Intrinsically conductive solutions involve well-recognize polymers such as PPy, P3HT, PEDOT:PSS, and PANi-CSA. Non-conductive solutions, instead, include insulating polymers that are straightforward to electrospun in combination with conductive species such as metals/metal oxides nanoparticles or other small molecules.

Conductive polymers have a low solubility in many solvents and are usually hard to electrospun due to this property; also, low molecular weight can inhibit fiber formation. Otherwise, the blending and encapsulation of conductive polymers/fillers such as carbon-based materials (graphene, graphene oxide), metallic nanoparticles, or metal oxides require a precise and accurate optimization of polymer solution characteristics and processing and environmental parameters. Furthermore, an incorporated filler with larger size and higher content will result in a greater reduction in permeability and mechanical performance, which restricts the materials’ application. This is mainly ascribed to the agglomeration of additives and heterogeneity, microstructure embrittlement and the discontinuity of electrospun nanofibers [55].

As well, even if C-SPs possess favorable electrical conductivities, they show often low mechanical properties, presenting a challenging option in some application fields as wearable electronics or sensors, for example. So far, various C-SPs, such as polyacetylene (PA), polythiophene (PT), polypyrrole (PPy), polyaniline (PANi), and poly [3,4-(ethylenedioxy)thiophene] (PEDOT) were used in coating certain layers; and even natural material fibers (i.e., cotton, wool, and silk), polymeric fibers (i.e., polypropylene (PP), polyacrylate (PA), polyethylene terephthalate (PET), polyurethane (PU), and polyacrilonytrile (PAN)) were utilized as substrates.

The electrospinning of conductive polymers as well the combination of insulating polymers and conductive species can be realized in different ways as detailed thereafter.

### 4.1. Blends and Composites

In the last decade, the physical mixing of biodegradable polymers with C-SPs phases was recognized as one of the most efficient strategies to fabricate blended or composite materials with improved electro-conductive functionalities. In particular, conducting polymers in combination with insulating ones can facilitate a homogeneous distribution of the electro-sensitive molecular groups and a more efficient transfer of electrical signals in different environmental conditions, which is suitable for a broad range of applications (i.e., biomedical, environmental, bio-sensing). This also depends on the versatility of the fabrication processes, which is based in turn on chemical or physical dispersion.

From this point of view, the processes can be classified in two different groups as a function involving solvent dissolution or polymer melting. Polymeric solution blends via melt processing are commonly used to design industry-scaled conductive systems. In this case, due to the conducting polymers’ mechanical dispersion in a thermoplastic melt, fully moldable and extrudable systems were produced, concurring with the development of percolative paths of conduction at lower C-SPs volume contents [56]. Meanwhile, all processes involving the dissolution of the polymer in various solvents are generally driven by specific chemical reactions, mainly requiring the group interaction of selected polymer chains to promote the solubility in organic solvents. In this context, the use of protonic acids such as dodecylbenzene sulfonic acid (DBSA), camphor sulfonic acid (CSA), or phosphoric acid diesters can be required to trigger the doping reaction that is used to activate the electro conductive sites into the final product [57].

As for the blending systems, various C-SPs have been successfully combined with other materials to satisfy a specific need and application. Polymers such as P3HT can be blended at different ratios to optimize the electrical and mechanical properties of the nanofibers. While the low molecular weight makes the fabrication of pure P3HT extremely difficult, a few methods such as assisting the extrusion by constantly covering the needle with solvent had worked tremendously to assist fiber formation; however, the solution concentrations are usually high (11–13%) and with fibers in the micro scale [58]. At lower concentrations such as 7 wt %, fibers could be formed with limited length and the presence of beads. Due to this behavior, a common approach is blending the conducting polymer with a secondary polymer to assist with the formation of nanofibers.

As for the composite materials, fine dispersions of PANI and/or PPy conductive fillers were largely investigated to address the limitations of non-polymeric conductive fillers (i.e., graphene, carbon nanotubes) during the process of insulating nanofibers in order to enhance the spatial distribution of conductive phases [59].

### 4.2. Coating the Nanofibers by C-SPs Thin Films

Among the most common techniques to create conductive nanofibers, coating electrospun nanofibers with conductive materials is widely reported in the literature. Compared to other techniques, it is easy to perform and permits obtaining fibers with excellent performances.

In order to form a conductive system based on coated electrospun nanofibers, a two-step process is required: first, the electrospinning of a polymer commodity or a weakly or non-conductive polymer but with other specific properties, then the realization of a conductive layer by one of the coating techniques, i.e., dip coating, spray coating, spin coating, drop casting, electrochemical deposition, physical or chemical vapor deposition, etc. In its turn, the coating step for some techniques can be divided into other two steps: (1) deposition of the conductive solution or of the precursors/oxidizing agent, and (2) drying or polymerization. The solvent evaporates during the drying process, depositing a conductive layer onto the fiber surface; during the polymerization step, instead, an in-situ polymerization of the physically adsorbed C-SPs is promoted. To achieve an increased conductivity, the coating step can be repeated multiple times. Tang et al. reported the preparation of highly stretchable carbon nanotube (CNT)/thermoplastic polyurethane (TPU) composite nanofiber yarn (CTY) through the dispersion of the reinforced CNTs into a flexible TPU matrix uniformly and electrospinning multi-needle liquid bath; then, CNT ink was dip-coated onto CTY to form the highly conductive coating. To improve the coating effect, DMF solvent was added to the CNT ink, and the conductivity of OCTY was increased by 5 orders of magnitude as compared to CTY. CNT at 3 wt % is the optimum dip-coating concentration for 1 min dip-coating time [60]. In another work, electrospun hyperbranched (HBPU)/linear polyurethane (LPU) nanofiber meshes were spray-coated with multiwalled carbon nanotubes (MWNTs). Observations revealed that the patterned-coated MWNTs looked different with an LPU/HBPU composition: the surface of nanofibers are well coated with MWNTs with an increase in HBPU content included in the nanofibers, whereas the MWNTs in the pure LPU nanofiber mesh had a well-networked MWNT structure across all of the nanofiber meshes, resulting in high conductivity for a pure LPU/MWNT nanofiber mesh. Some reports have emphasized that increasing the spray frequency can lead to a decrease in the electrical resistance and improve surfaces for biosensing applications [61]. An example of an in-situ modification and self-assembly is presented for silver nanoflowers (AgNFs). By increasing the modification times during an in situ self-assembly, the surfaces of nanofibers are gradually covered by AgNFs, and surface-connected AgNF petals form a dense shell network of conductive AgNFs. The rigid ACNTs and AgNFs enhance the tensile strength of TPU/ACNT/AgNF at a strain of 0–400% and maintain the elongation at composite break. Additionally, the dual conductive networks provide great conductivity to the M-CNCs as well as enhanced antibacterial property and improved strain sensing [62].

Another approach includes the coating of nanofibers with the conducting polymer in the form of a film. In this method, the nanofibers are primarily fabricated by the electrospinning technique, and then, these are coated via thin film or in situ polymerization. An excellent example includes the fabrication of PANI-coated PU fibers for potential vascular tissue engineering. The PU fibers were prepared via electrospinning and conditioned by soaking the formed fibers in ethanol, enhancing the hydrophobicity of the surface and therefore improving the polymerization of PANI. The as-spun fibers had an average diameter of 1.45 µm, while the fibers with the PANI coating had an average diameter of 1.64 µm. The composite fibers demonstrated an electrical conductivity of 4.66 × 10^−2^ S/cm. The composite showed a slight anticoagulant effect with enhanced cell adhesion and activity of the endothelial cells [63].

In this context, the combination of electrospinning with polymer deposition techniques (i.e., spin coating, solid-state polymerization) may be successfully used to realize innovative substrates with more complex architectures and electroconductive coatings.

### 4.3. Coatings by Electrodeposition

Nanofiber coatings have a number of unique properties, making them useful in a plethora of applications. They are extremely lightweight and strong and have a high surface area-to-volume ratio, which makes them highly effective at absorbing or releasing heat and moisture. They also have excellent electrical and thermal conductivity and can be used to enhance the performance of a wide range of materials and products. One technique to form a conducting nanofiber is by coating a precursor nanofiber with the C-SP. This action can occur at the tip of the needle by using a coaxial set-up, by thin film addition, or by electrostatically adhering the molecule of interest. An interesting approach useful to realize this kind of coating is the use of electro-fluid dynamic techniques (EFDTs): namely, electrospinning, electrospraying and electrodynamic atomization.

**Figure 3:**
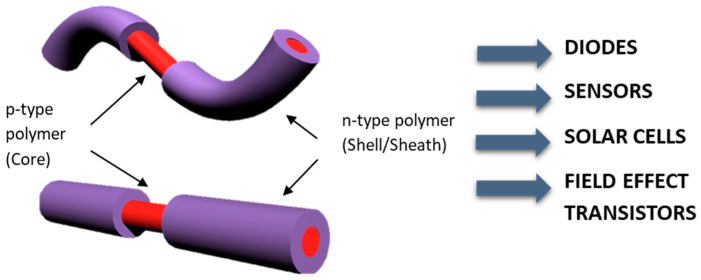
P- and n-type coaxial nanofiber semiconducting polymers formed as a nanofiber provide an opportunity for the formation of a p-n junction that can be chained for electroactive textiles and single nanofiber devices. Reprinted from [64].

**Figure 4:**
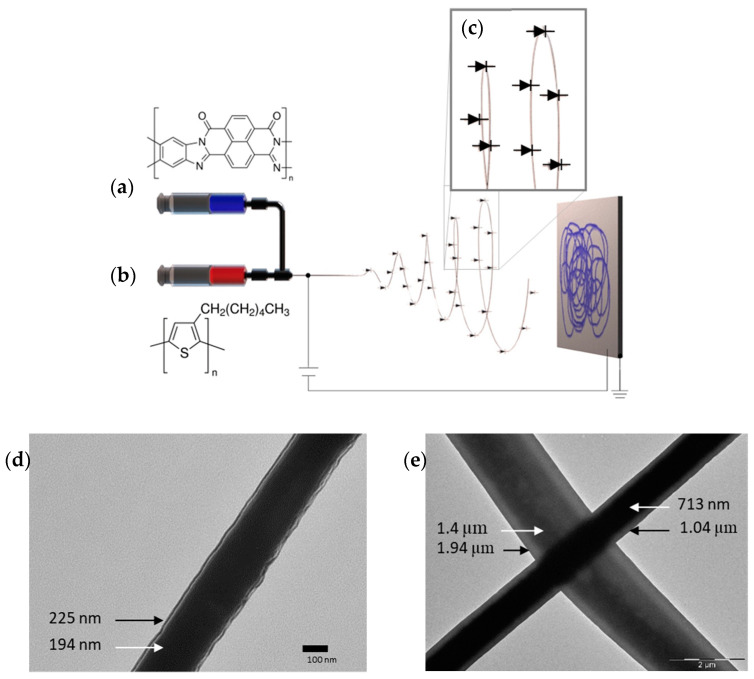
Fabricated electrospun continuous coaxial nanofiber–p-n junctions. The shell/sheath is formed by the BBL solution (**a**, blue) and covers the P3HT/PS solution (**b**, red) core. An electric field overcomes the surface tension, forming nanofibers. Inset: (**c**) diode symbols representing the continuous heterojunction formed between the core and the shell. (**d**,**e**) Formed coaxial nanofibers where BBL covers the P3HT/PS core. Adapted with permission from [64].

An example is coating the nanofiber by a coaxial-needle set-up (Figure 3). Polymers such as the n-type ladder BBL could be challenging to process by itself in the electrospinning technique; however, it could be straightforward when a supportive polymer is used in the process. In this case, a combination of P3HT and PS were used as the supportive polymer. In this specific case, the PS supports the formation of P3HT fibers by mixing it together, while the PS/P3HT composite supports the formation of the BBL film to cover the core fiber, forming a coaxial structure (Figure 4) [64].

**Figure 5:**
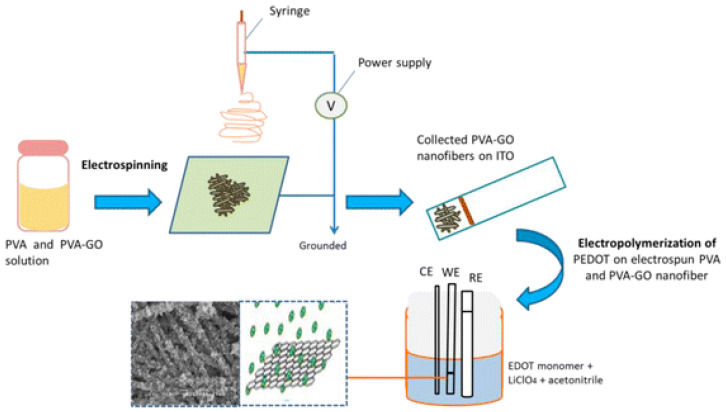
Schematic diagram illustrating the steps involved in the fabrication of PVA-GO/PEDOT nanofibers prepared by combining electrospinning and electropolymerization methods. Adapted with permission from [65]. Copyright 2019 American Chemical Society.

Another example reports the realization of a PVA-GO composite by electrospinning and covering with PEDOT by electropolymerization (Figure 5). The applied voltage and the concentration of GO was tailored in order to form smooth and uniform smaller diameters. The electropolymerization was performed by applying 1.2 V of electrodeposition potential for 5 min with effective growth over the nanofibers mesh [65].

## 5. Challenges of Electrospinning C-SPs

The applications of C-SPs for the fabrication of electroconductive fibers for new devices and applications has grown in the past years; however, there still some challenges that need to be overcome to enhance and promote the reliability of the electronic properties of such polymers. These challenges involve preparation, processing, and costs. One of the main challenges is the high viscosity of polymer solutions with conducting polymers, which makes it difficult to achieve uniform and consistent fibers. This can lead to variations in fiber diameter and morphology, which can affect the properties of the resulting fibers. Additionally, conductive polymers tend to degrade or lose their conductivity under the high electric fields used in electrospinning, which can further complicate the process.

Another challenge is the lack of suitable solvents for some conductive polymers, which makes it difficult to prepare spinnable solutions. Additionally, incorporating conductive fillers, such as carbon nanotubes or graphene, into the electrospun fibers can also be difficult due to aggregation. The cost of some conductive polymers is also a concern, as it can make it difficult to produce electrospun fibers in large quantities at a low cost and with extended shelf life.

Furthermore, there is still a lack of understanding about the properties and behavior of electrospun conductive polymers fibers, which makes it difficult to optimize the electrospinning process and control the properties of the resulting fibers when performed under uncontrolled environmental conditions. This lack of understanding has hindered the widespread use of conductive polymers in electrospinning. Despite these challenges, researchers are continuing to work toward developing strategies to overcome these issues and make conductive polymers more viable for electrospinning at room temperature.

## 6. Applications

Electrospinning is a widely used technique for the production of nanofibers, which are thin fibers with diameters in the nanometer range. These fibers have high surface-to-volume ratios, meaning they have a large surface area relative to their size, as well as high porosity, or the amount of empty space within the material. These properties give nanofibers unique characteristics that can be exploited for a variety of applications. One of the key benefits of electrospinning is the ability to use a wide range of polymers to create nanofibrous structures as shown above. This allows for the production of materials with specific properties that can be tailored to meet the needs of a particular application.

One interesting feature of electrospun fibers is that the conductivity of the polymer solution used to create them can affect their size. When conducting polymers are electrospun alone or in combination with another polymer, the higher conductivity of the polymer solution often leads to the production of thinner fibers. This is because the electrical forces acting on the polymer solution during the electrospinning process are stronger, leading to a more focused and concentrated stream of material that is drawn out into thinner fibers.

### 6.1. Electronics

**Figure 6:**
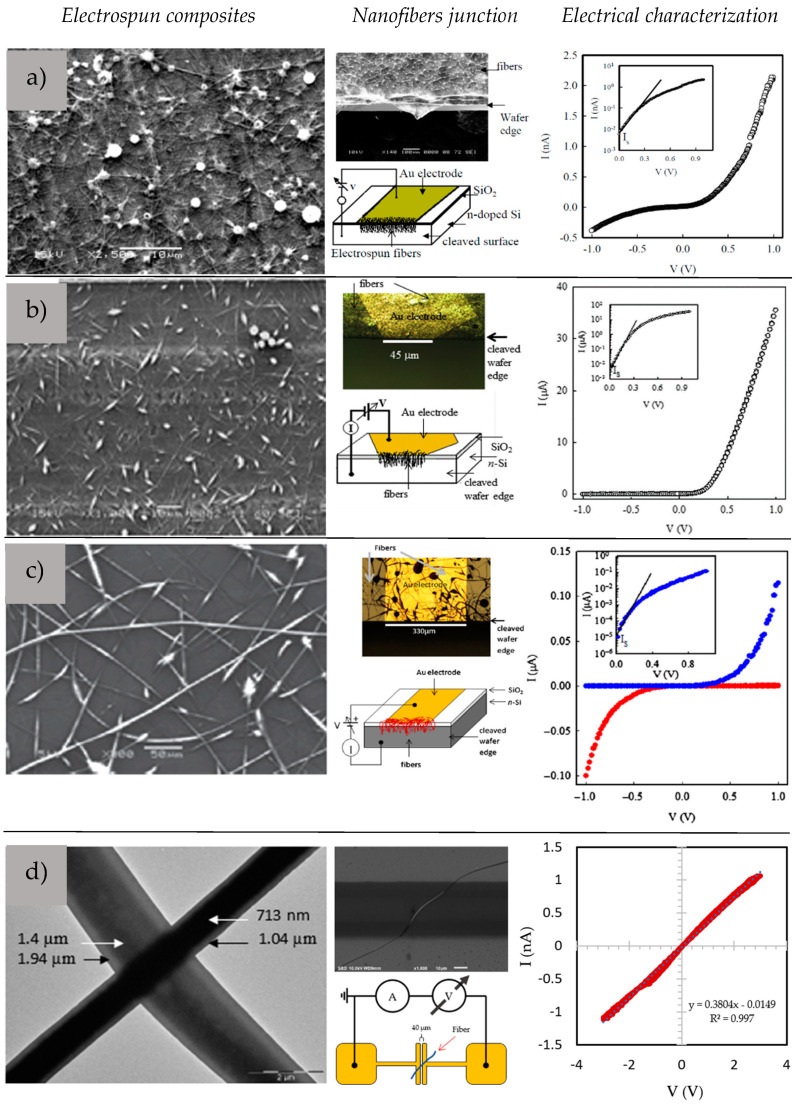
Semiconductive composite nanofibers. PVDF-TrFE/PEDOT (**a**), PLA/PANI (**b**), PLA/P3HT (**c**), and core–shell P3HT/BBL (**d**). Adapted with permission from [64,66,67,68].

Fiber-based devices [66,67,68], structures [2], and wearables [1,2,17] have been formed using the electrospinning technique. Nanofibers with specific electrical properties have been applied to fabricate basic electronic components such as diodes. In this case, C-SPs were electrospun over doped silicon, forming a heterojunction. PVDF-TrFE with PEDOT-PSSA composite nanofibers were spun over a doped silicon substrate. The integration of PEDOT-PSSA enhances the charge in solution and facilitates the formation of uniform nanofibers at low polymer concentrations in DMF, eliminating the beading effect commonly observed in solutions with high polymer concentrations. These nanofibers at the interface of the n-type Si led to the formation of a Schottky diode with a turn-on voltage of 0.52 V, with an ideality parameter of 3.2, and a barrier height of 0.65 eV (Figure 6a) [66]. Another composite such as PANI-CSA/PLA nanofibers was also prepared from low concentrations of PLA in CHCl3 with uniform incorporation of the two polymers. Nanofibers formed with concentrations as low as 1 wt % lead to the fabrication of fibers with diameters ranging from 10 to 300 nm. The formed diode had a low turn-on voltage, a high rectification ratio with an ideality parameter of 1.6, and a barrier height of 0.49 eV (Figure 6b). The diode was able to rectify low-frequency AC signals with moderate efficiency when connected in a half-wave rectifier circuit [67].

Another successful implementation of the electrospinning included PLA/P3HT composite nanofibers with low PLA concentrations. The traditional electrospinning method was unable to form pure PLA fibers at 5 wt % in CHCl3; however, the addition of P3HT in the solution made it possible to create composite fibers that possess electrical conductivity. As the concentration of P3HT in the solution increases, the formation of well-formed fibers also increases. These fibers possess diameters ranging from 100 nm to 4 μm and exhibit electroactivity (Figure 6c). By using an n-doped Si/SiO2 substrate with pre-patterned Au electrodes, a p-n diode was fabricated and tested, which also exhibited the ability to sense UV radiation and maintain functionality with an increase in on/off ratio and a decrease in turn-on voltage [68]. This breakthrough extends the range of applications of this biocompatible and biodegradable polyester to include electronic devices such as diodes and sensors with reduced toxicity.

### 6.2. Bio-Filtration

Biofiltration is a natural approach which requires the use of a bioreactor containing living material to trap and naturally decompose contaminants [69,70]. This technique is widely used in air and water treatment and has highly benefited from the fabrication of nanofibers that can synergistically be embedded with living material or perform the filtration process independently of it [71]. There are several ways in which nanofibers can be used in biofiltration systems. Nanofiber membranes can be used as a substrate for biofilms, which are thin layers of microorganisms that adhere to a surface. The microorganisms in the biofilm can then remove pollutants from the water or air that passes through the membrane [72,73]. Nanofiber filters can be used to remove pollutants from water or air by using microorganisms that are embedded in the fibers. The microorganisms can then break down the pollutants into less harmful substances [74]. Nanofiber scaffolds can be used to support the growth of microorganisms that are involved in the biofiltration process [75]. The scaffolds provide a surface for the microorganisms to attach to, which can help improve their effectiveness at removing pollutants. In general, the use of nanofibers in biofiltration systems can help to improve the efficiency of the filtration process and remove a wide range of pollutants, including pathogens, organic compounds, and heavy metals.

**Figure 7:**
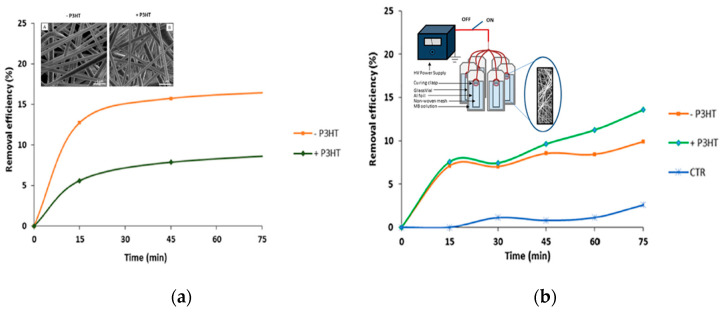
PVDF-TrFE/P3HT composite nanofibers for molecular adsorption. Inset (**a**) Unloaded (−P3HT) (A) and loaded (+P3HT) (B) nanofibers without (**a**) and with (**b**) the application of an external electric field. Inset (**b**): Schematic of the nanofibers system for controlled adsorption. Adapted with permission from [76].

Fibrous structures could enhance the selective absorption of certain molecules in order to facilitate its removal. The capability to combine polymers for a nanofiber fabrication step has advanced new directed applications. One example includes the usage of piezoelectric polymers such as PVDF-TrFE embedded with the semiconductor P3HT as a nanofiber mesh. It has been shown to improve the adsorption of the negatively charged molecule methylene blue (MB) by applying an external voltage on the PVDF/P3HT nanofibers. These composite nanofibers offer various advances such as a high surface-to-volume ratio that enhances the active surface area available to adsorb the molecule and the electric field applied to improve the molecular pooling of PVDF during the spinning process due to the piezoelectric moment which creates a dipole moment. Furthermore, an external voltage can be applied to the fiber mesh which helps the interaction of MB toward P3HT and therefore influences the adsorption of MB molecules (Figure 7b) [76].

Structural fine tuning of the nanofibers mesh pore size is also possible with the combination of C-SPs such as PEDOT embedded with nitrile butadiene rubber (NBR) and poly(ethylene glycol)dimethacrylate (PEGDM). In this case, PEDOT is synthesized in the fibers that contain EDOT. This process is known as oxidation, which promotes the polymerization of EDOT into PEDOT. In this case, it was completed by immersing of the swollen nanofibers in an aqueous solution of 1.5 M FeCl3 [77].

### 6.3. Tissue Engineering

Tissue engineering is another area where the electrospinning technique bloomed from the material used to the way the technique is implemented. While bone scaffolds and wound-healing applications still being researched and constantly advancing in their applications, a relatively new area that is being benefited by the fabrication of nanofibers is nerve and neural tissue engineering.

Some of the fibers could be coated by the material of interest such in the case of cellulose fibers coated with C-SPs to facilitate electrical stimulation on rat adrenal pheochromocytoma cells (PC12 cells). The fabrication of these cellulose scaffolds was mainly used to study nerve cell growth by using 0.05 M PNVPY and 0.15 M for P3HT. Coating the cellulose fibers with PNVPY showed the aggregation of nanoparticles, while the P3HT performed a coating without such aggregations. The addition of this coating also increased the nanofiber final diameter, which was more suitable for cell adhesion. The excellent biocompatibility of the composite led to the PC12 cells to adhere and proliferate on the fiber matt while preserving their original phenotype [78].

Another advantage of the C-SPs is the ability to fine-tune the conductivities by wet chemistry in a fast and reliable manner. In this case, we have the formulation of P3HT/PLGA, whose conductivity was fine-tuned by ferric chloride (FeCl3) doping. This doping procedure was performed by immersing the composite material in an ethanol solution of FeCl3 (10 mmol/L) at specific time steps. This composite resulted in the expected nerve cell proliferation while the doped composite, with increased conductivities, was able to promote the proliferation of the rat adrenal pheochromocytoma cells (PC12 cells). By inducing a nerve growth factor (NGF), the composite effectively promoted the growth and elongation of PC12 synapses. The increased conductivities up to 10^−2^ S/cm promoted the synaptic length over 40 µm, enhancing possible applications on nerve repair. Indeed, by fine tuning the conductivity of the composite, it can promote the high expression of Synapsin 1 (SYN1) and microtubule-associated protein 2 (MAP2), which is an important aspect in nerve repair and regeneration [79].

The optical properties of P3HT have been used in conjunction with other polymers by decorating or coating the surface of the fibers to make a heterojunction membrane. In this case, composite nanofibers of PCL-P3HT were coated with PPY by taking advantage of the photovoltaic properties of P3HT and the conductivity of PPY, which also enhances the system as an antioxidant. This composite demonstrated to promote PC-12 neurogenesis, growth, and survival. The coating procedure was performed by immersing the PCL-P3HT membranes in deionized water to provide a hydrophilic surface for in situ pyrrole polymerization. Subsequently, the meshes where immersed in an aqueous solution of pyrrole and ferric chloride (FeCl3) at a 1:1 volumetric ratio [80].

Recently, a large variety of composite fibers including PANI (an intrinsically conductive polymer characterized by recognized biocompatibility) has been investigated for the fabrication of electroactive devices for tissue engineering (Table 2). 

**Figure 8:**
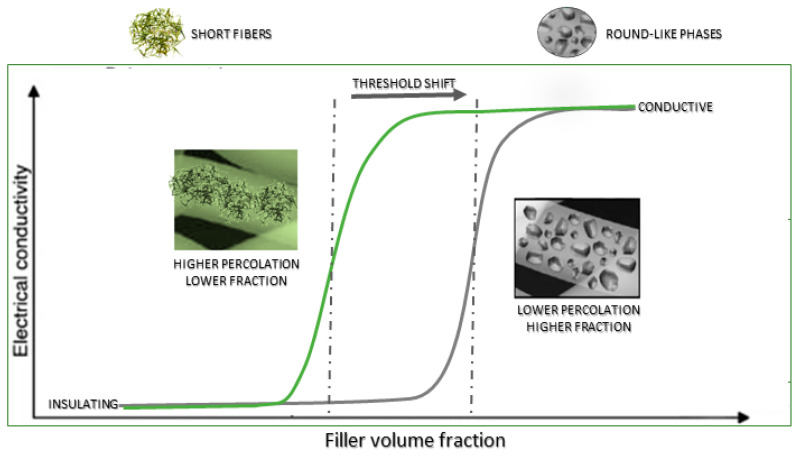
Schematic description of shape/volume fraction effect of ultrafine short fibers on the transfer mechanisms of electrical signals for in vitro cell interactions. Adapted with permission from [88].

Among them, researchers recently proposed a new formulation based on the dispersion of ultrafine polyaniline short fibers (sPANI) in a PCL matrix for the realization of electro-sensitive platforms for brain and cardiac tissue engineering. In this case, electrical conductivity is generated by the peculiar shape of the fillers, i.e., needle-like crystals, obtained by in situ precipitation [86]. This reaction (initiated by selected acid compounds) allows switching the initially non-conductive polymer toward an electro-conductive state by increasing the protonation degree via acidic dopant treatments that allow converting imine groups of the emeraldine base to iminium [87]. Starting from this evidence, it has been proved that ultrafine polyaniline short fibers (sPANi) dispersed in PCL solution and, then, processed via electrospinning, allow fabricating composite fibers with improved charge transport, in comparison with that of emeraldine base polyaniline (EB-PANI), due to the peculiar shape of fillers and their spatial distribution, which allow forming a percolative pathway (a mandatory condition to trigger the transfer of electrical signals) by reducing the filler amount used (Figure 8) [88].

Accordingly, Borriello et al., an efficient entrapment of sPANi short fibers into PCL electrospun fibers enables creating scaffolds able to stimulate myoblasts—that are myocardium cells—to accelerate the production of extracellular matter, thus supporting in vitro regeneration of the cardiac muscle [89]. More recently, Saracino et al. also demonstrated a relevant role of sPANI embedded into bioactive electrospun fibers on the structural and functional properties of primary astrocytes for brain regeneration. In particular, composite fibers including sPANI short fibers allow, firstly, to support the adhesion and growth of astrocytes over time; secondly, they induce the formation of actin filaments promoting a more efficient cytoskeleton rearrangement and cell alignment without a significant decay of active/passive bioelectrical properties of the astrocytes [90].

In the work of Nahra et al., three polymers, region-irregular poly(3-hexylthiophene) (P3HT), poly(N-vinylcaprolactam) (PNVCL), and with n-butyl acrylate (P(NVCL-co-ABu)), were synthesized and blended with PT using casting and electrospinning processes. The P(NVCL-co-ABu) blend improved the morphology and decreased the diameters of the electrospun fibers compared to PNVCL due to the presence of flexible segments of ABu. The entire mats produced in this study were found to be non-toxic. Specially, the blend of P3HT and P(NVCL-co-ABu) had a low contact angle value and low conductivity but showed the ability to promote cell proliferation and may be suitable for tissue regeneration due to the material’s geometry, wettability, and the small amount of iron ions released by P3HT [91].

Another polymer which has demonstrated excellent applications on neural tissue engineering is PEDOT:PSS. In this case, the use of cross-linked PEDOT:PSS with 3-glycidoxypropyltrimethoxylane (GOPS) and covered with laminin was successfully used by applying a pulsed signal to enhance the elongation of neural stem cells, which impacted on the cell differentiation at higher percentages of neurons and provided higher elongations and longer neurites when cultivated under an electrical pulse. In this work, not only the cross-linked PEDOT:PSS was confirmed to be non-cytotoxic but also the well-known polymers such as P3HT, F8T2, and MEH-PVV were also demonstrated to be viable polymers to such applications with non-cytotoxic effects. In this work, thin films were used as the choice structure due to their straightforward fabrication, which were coated with laminin to promote cell adhesion; however, the polymers could be used for the fabrication of fibrous structures promoting even more the differentiation and elongation of the neural cells. The PEDOT:PSS thin films showed a conductivity of 5.8 Ω^−1^ m^−1^. The electrical simulation was performed by applying an electrical pulse with a magnitude of 1 V to gold electrodes 1 cm apart. The formed electric field is therefore said to steer laminin toward the surface of the film, which promotes their binding to the cells integrins. The conductive layer had a positive effect on the elongation differentiation and neurite outgrowth. The cell behavior under the effect of the electric field promoted the elongation and stretch of the cells with an aspect ratio of ca. 0.5 in comparison with those without the electrical stimulation, which retained an aspect ratio of ca. 0.2. On the other hand, the aspect ratio of the cells under the electrical stimulation was significantly lower which relates to longer neurons, therefore validating the positive effect on neural stem cells differentiation. Lastly, the neurites were significantly longer under electrical stimulation than those with no electrical stimulation leading to an average length of 108 µm and 73 µm, respectively [92].

**Figure 9:**
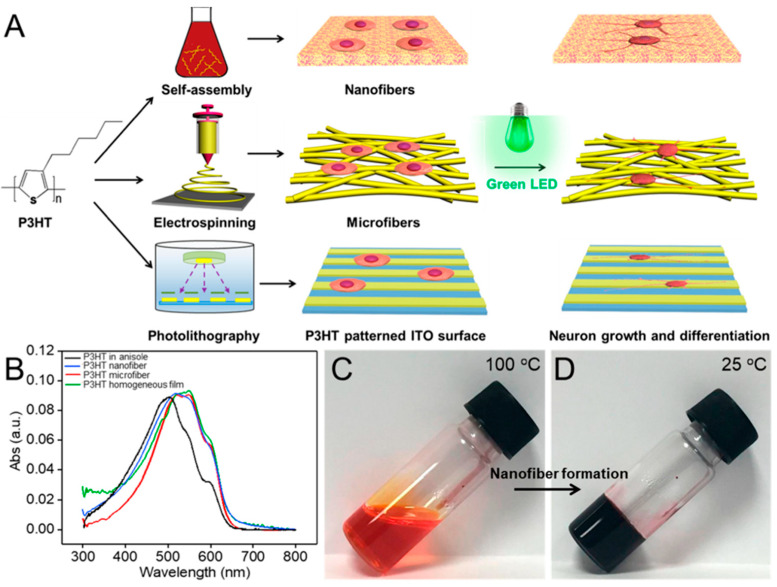
(**A**) Schematic illustration of the preparation of P3HT micro/nanofibers and P3HT-patterned surface for PC12 cell culture. (**B**) UV-vis absorption spectra of P3HT polymer in anisole solution, P3HT micro/nanofiber, and homogeneous films. (**C**,**D**) Digital photographs of P3HT in hot anisole (100 °C) (**C**) and after being cooled to 25 °C (**D**). Reprinted with permission from ref. [93]. Copyright 2022 American Chemical Society.

Using P3HT to has proven to be an excellent semiconductive polymer to promote the adhesion, further differentiation, and growth or neural cells under green LED irradiation. The promising optoelectronic properties and crystalline formation due to strong molecular π–π interactions has proven to be an option to promote the photostimulation of neurons due the generation of photocurrent. Furthermore, the topography of this material also enhances several regions of the neural growth such as branches and neurites. By fine tuning the fabrication of self-assembled nanofibers and electrospinning microfibers, researchers have shown that neurons, under photostimulation, enhance the formation of neural branches with dendritic axons sprouting out from the cell soma when there is growth over P3HT nanofibers of 50 nm. Meanwhile, neurons growth over electrospun fibers of about 1 µm of diameter enhanced the formation of longer and thinner neurites. The microfibers in question were prepared by mixing P3HT and PCL/THF (12 w/v%) to form a solution with a weight ratio of 1:1. The fibers were electrospun at room temperature by using a high voltage of 8 kV with a tip-to-collector distance of 12 cm and a flow rate of 1 mL/hr, and they were collected over silver paper. While films were also compared in this work, it was shown that the morphology of the P3HT film affects cell differentiation with the fiber structures improving cell adhesion and differentiation and providing an effective surface to enhance nerve regeneration (Figure 9) [93].

### 6.4. Biosensors

Nanofiber biosensors are devices that use nanofibers to detect the presence of specific biological molecules or substances in a sample. These sensors are typically made by electrospinning a polymer solution that contains a biologically active material, such as an enzyme or antibody, onto a substrate. The nanofibers are then collected on the substrate to form a coating, which can be used to detect the presence of the target molecule. Indeed, nanofiber biosensors are highly sensitive and specific, and they can be used to detect a wide range of biological molecules and substances, including proteins, hormones, and pathogens.

There are several ways in which nanofiber biosensors can be used to detect biological molecules, including colorimetric, enzyme targeting, electrochemical, fluorescence detection, and volatile organic compounds [94,95,96,97].

Bhattacharjee et al. used the interaction between nucleocapsid and spike proteins to detect SARS-CoV-2 by colorimetric means. Polydiacetylene (PDA) nanofibers were created through a chemical process called functionalization, using a compound called PCDA monomer and the electrospinning. These fibers were designed to bind to a specific protein called nucleocapsid antibody (anti-N) found in SARS-CoV-2. The anti-N protein was then attached to the functionalized PDA fibers by conjugation, resulting in PU-PDA-NHS-anti fibers. When these PU-PDA-NHS-anti fibers were exposed to the S protein of SARS-CoV-2 at room temperature, they underwent a color change from blue to red, indicating that they could be used as a method for detecting SARS-CoV-2 through a colorimetric reaction [98].

Similarly, enzyme-linked immunosorbent assay (ELISA) technique uses enzymes and antibodies to detect the presence of specific molecules. The nanofiber coating is functionalized with an enzyme or antibody that specifically binds to the target molecule. When the target molecule is present in the sample, it binds to the enzyme or antibody, and the resulting reaction produces a detectable signal [99]. Fiber-based ELISA using nanofibers of poly L-lactic acid (PLLA) and cellulose acetate (CA) were compared with cotton fibers to detect the level of C-reactive protein (CRP). The limit of detection of CRP of 13.00 pg/mL for PLLA nanofibers, 53.00 pg/mL for CA nanofibers, and 27.32 pg/mL using cotton microfibers. This fiber-based ELISA can also be used to test for CRP in saliva, which could potentially help with early detection of certain diseases like cardiovascular disease and infectious diseases. Additionally, this method is relatively inexpensive, easy to use, and can be done at home using cotton swabs with a high-throughput colorimetric screen. However, the results showed that the immobilization of antibodies on PLLA nanofibers was more stable compared to immobilization on CA and cotton fibers [100].

The electrochemical detection technique uses an electrode coated with a nanofiber layer that contains an enzyme or other biologically active material. When the target molecule is present in the sample, it reacts with the enzyme or other biologically active material, which generates an electrical current that can be measured and used to detect the presence of the target molecule [101,102,103]. The fluorescence detection technique uses fluorescent molecules or dyes that are incorporated into the nanofiber coating. When the target molecule is present in the sample, it can cause a change in the fluorescence of the coating, which can be used to detect the presence of the target molecule [104]. The usage of polymers such as PANi in electrochemical and fluorescent biosensors has also been studied. For example, electrochemical and fluorescent dopamine biosensors based on PANi/CQDs composites were studied for the detection of low concentration of dopamine. The PANi/CQDs composite was electrospun and achieved an average diameter of 320 nm. An electrochemical biosensor was developed using an electrospun film made of a composite of PANi and CQDs on an FTO substrate. This biosensor was able to detect dopamine using cyclic voltammetry and amperometry at various concentrations ranging from 1 to 100 μM. The electrospun PANi/CQDs fiber film exhibited good selectivity and high sensitivity toward dopamine. Furthermore, a fluorescent biosensor was also developed using a PANi/CQDs solution to detect various concentrations of dopamine (10 nM to 100 μM) through fluorescent quenching. The PANi/CQDs composite showed good sensitivity toward dopamine sensing and may be a promising candidate material for the sensitive and selective detection of dopamine, which could be used as a sensing platform in future sample analysis [105]. Likewise, Liu et al. developed a straightforward approach to assemble DNA-functionalized gold nanoparticles on nanofibers. By constructing the membrane toward the detection of the BRCA I gene fragment which is related to breast cancer, the sensor showed high sensitivity and good selectivity in a reproducible manner [106].

**Figure 10:**
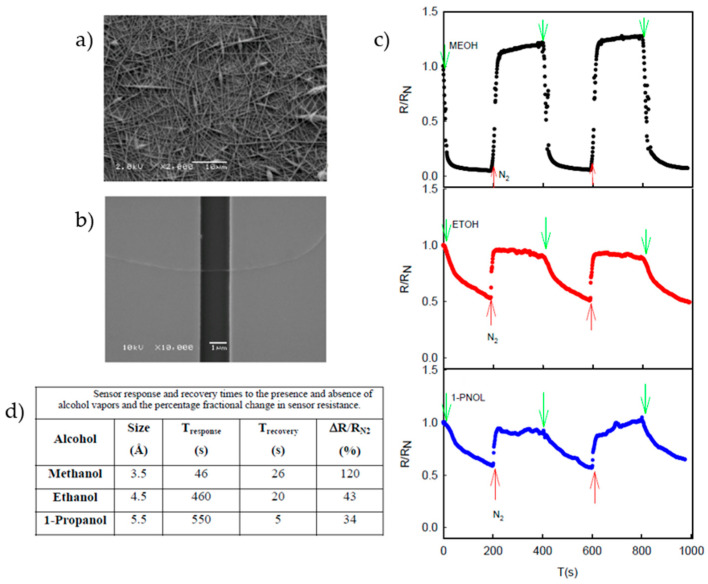
SEM images of 3 wt % PLA/PANI-CSA (**a**) and single nanofiber sensing device (**b**). Sensor characterization of alcohol vapors (**c**). Response and recovery times for each alcohol (**d**). Adapted with permission from ref. [107].

Another application of the electrospun nanofibers of PANi blended with PLA was studied as sensors for detecting aliphatic alcohol vapors (Figure 10). Aliphatic alcohols are a class of compounds that includes straight-chain and branched-chain alcohols, and they are commonly used as solvents and intermediates in various industrial and consumer products. The ability to detect these vapors is important for a variety of applications, including environmental monitoring and industrial safety. The researchers used electrospinning to fabricate PLA/PANI nanofibers with different ratios of PLA to PANI. They then tested the ability of these nanofibers to detect aliphatic alcohol vapors of varying sizes, including methanol, ethanol, and propanol. They found that the nanofibers were able to detect all the alcohol vapors tested, with the highest sensitivity observed for methanol. The sensitivity of the nanofibers increased with an increase in the PANI content, indicating that the PANI component played a significant role in the sensing performance. Overall, the results of this study suggest that electrospun PLA/PANI nanofibers can be used as sensitive and selective sensors for detecting aliphatic alcohol vapors. The high sensitivity and selectivity of these nanofibers, as well as their low cost and ease of fabrication, make them attractive for use in a variety of sensing applications [107].

## 7. Conclusions and Future Trends

As we move into the future, the utilization of conductive polymers in electrospinning will play a vital role in the advancement of technology. Electrospinning, the process of producing ultra-fine fibers by subjecting a polymer solution to an electric field, will be revolutionized by the integration and applications of C-SPs. However, as with any new technology development, there will be obstacles to overcome. Here, we have described the importance and versatility of C-SPs nanofibers. Understanding the variables and properties the electrospinning technique offers for the fabrication of nanofibers is of key importance for the improvement and applications of C-SPs in numerous research and development areas. In the formed structures, the conductive polymers are benefited by the high surface-to-volume ratio and directionality the fibrous structure brings. By this, the molecular reorganization into the fiber improves the addition or extraction of electrons from the delocalized π-bonded polymer backbone, promoting the formation of charge carriers such as polarons, bipolarons, and solitons under various intrinsic and external stimulation. The fibers showed conductivities that could be fine-tuned by controlling the doping process. The formed devices and structures were able to achieve remarkable outcomes in tissue engineering for neural nerve cells and other applications that required an electroactive component, making it evident that the utilization of the electrospinning technique will impact the application of new C-SPs. However, as we move forward, researchers are developing new technologies that will overcome these obstacles. With new understanding of the properties and behavior of electrospun conductive polymers fibers, we will be able to optimize the electrospinning process and control the properties of the resulting fibers, making conductive polymers more viable for electrospinning toward a more bio sustainable approach [108]. In this view, the use of biopolymers as promising eco-friendly, economical, and energy-efficient materials is rapidly emerging for environmental and healthcare monitoring. Several studies recently suggested to process polysaccharide, polypeptides, or polyphenols by electrospinning to design a wide range of biosensors for different uses [109,110,111,112,113,114,115,116]. Moreover, recent studies have also demonstrated that recently discovered biopolymers with interesting electroconductive polymers (i.e., polydopamine [117], eumelamins [118]) can be easily adapted to the electrospinning process, paving the way for a new era of fibrous platforms with tunable electrical conductive/semiconductive properties with improved biosustainability and limitless possibilities of applications in different fields.

## Figures and Tables

**Table 1:** Common C-SPs for the electrospinning of nanofibers.

Polymer	Chemical Structure	Chemical Formula
Polyacetylene (PA)	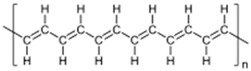	(C_2_H_2_)_n_
Polypyrrole (PPy)	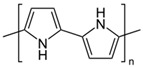	H(C_4_H_2_NH)_n_ H
Polyaniline (PANI)	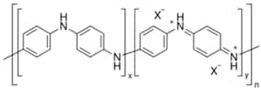	([C_6_H_4_NH]_2_ [C_6_H_4_N]_2_)_n_
Poly(3-hexylthiophene-2,5-diyl) (P3HT)	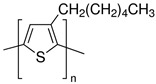	(C_10_H_14_S)_n_
Poly(3,4-ethylenedioxythiophene) (PEDOT)	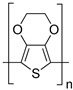	(C_6_H_4_O_2_S)_n_
polythiophene (PT)	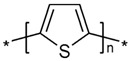	(C_4_H_2_S)_n_
poly(p-phenylene vinylene) (PPV)	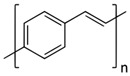	(C_8_H_6_)_n_
Poly(benzimidazobenzophenanthroline) (BBL)	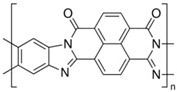	(C_20_H_6_N_4_O_2_)_n_

**Table 2:** PANI-based composites fibers as functional substrates for tissue engineering.

Host Polymer for PANI [Ref.]	Conductivity (σ)	Mechanical Properties	Biological Functionalities
PLCL [81]		E = 50 MPa	Fibroblast adhesive
	13.8 mS/cm	εr = 207.85%	Metabolism promoter
		UTS = 0.69 MPa	
Gelatin [82]		E = 1384 MPa	Influence smooth muscle-like morphology (i.e., microfilaments)
	17 mS/cm	εr = 9%	
		UTS = 10.49 MPa	
PLGA [83]	3.1 mS/cm	E = 91.7 MPa	Cardiomyocite marker overexpression of (Cx43) and (cTnI)
PCL [84]	63.6 mS/cm	E = 55.2 MPa εr = 38% UTS = 10.5 MPa	Myotube formation
Collagen/HA [85]	2 mS/cm	E = 0.02 MPa εr = 78% UTS = 4 MPa	Mineralization
